# Developmental Changes in Mental Rotation: A Dissociation Between Object-Based and Egocentric Transformations

**DOI:** 10.5709/acp-0187-y

**Published:** 2016-06-30

**Authors:** Sandra Kaltner, Petra Jansen

**Affiliations:** Department of Sport Science, University of Regensburg, Germany

**Keywords:** mental rotation, response inhibition, developmental changes, object-based and egocentric transformations

## Abstract

The present study was conducted to investigate developmental changes of mental rotation performance. We compared children, adults, and older adults regarding their performance in object-based and egocentric transformations. Both children and older adults showed higher overall reaction times compared to adults. Results were interpreted against the background of impaired working memory capacity in both children and older adults. Since mental changes in working memory are mediated by age differences in cognitive processing speed, cognitive speed is supposed to be the underlying factor. Regarding both types of transformations, an advantage of egocentric over object-based human figures was only found in adults which led us to tentatively propose that children and older adults show deficits in perspective taking compared to adults.

## Introduction

 Mental rotation (MR) is a specific visuo-spatial ability which involves the process of imagining how a two- or three-dimensional object would look if rotated away from its original upright position (Shepard & Metzler, 1971). In the classic paradigm of Cooper and Shepard (1973) two stimuli are presented simultaneously next to each other on a screen and the participant has to decide as fast and accurately as possible if the right stimulus, presented under a certain angle of rotation, is the same or a mirror-reversed image of the left stimulus, the so called *comparison figure*. While angular disparities are varied systematically, response times, accuracy rate, and MR speed are serving as dependent variables. 

 In MR there are two different classes of mental spatial transformations: object-based and egocentric transformations (Zacks, Mires, Tversky, & Hazeltine, 2002). Whereas in egocentric transformations participants mentally change their own perspective and thus imagine rotating their own body in order to make a decision, in object-based transformations the observer’s position remains fixed and participants mentally rotate the object in relation to the surrounding environment (Devlin & Wilson, 2010; Jola & Mast, 2005). 

 Regarding developmental changes, Vandenberg and Kuse (1978) found large age differences in mental rotation performance. However, the investigation of developmental changes is limited by two facts: 1) Many studies have limited their efforts to one transformation type (e.g., egocentric: Estes, 1998; object-based: Marmor, 1975), and 2) literature is focused on one or two age groups (e.g., children: Piaget & Inhelder, 1971; adults: Wraga, Creem, & Proffitt, 2000; older adults: Jansen & Kaltner, 2014). So far the comparison of these two types of transformations (object-based vs. egocentric) with more than two age groups trough a unique design has not been conducted. This motivated us to compare three different age groups (children, adults, older adults) regarding their performance in object-based and egocentric transformations using a standardized design. 

### Object-based and egocentric mental transformations

 The use of each transformation depends on the type of judgment that has to be made: An object-based transformation is elicited by a task where two images are typically presented simultaneously side-by-side and participants are required to perform a same-different decision by judging whether the right stimulus is the same or a mirror-reversed (different) version of the left stimulus. An egocentric transformation is generally induced by the presentation of a body stimulus, for example, a single human figure raising one arm (left or right), presented under different orientations. The participant has to decide which arm is raised resulting in a left-right judgment (Steggemann, Engbert, & Weigelt, 2011). 

 Regarding object-based transformations reaction times (RTs) typically show a linear increase with increasing angular disparity between the two presented objects (Shepard & Metzler, 1971). The authors interpreted the linear relationship in object-based transformations as a hint that the process of mentally rotating an object is analogous to the manual rotation of an object. However, in egocentric transformations RTs only start to increase at angles above 60° and 90° (Keehner, Guerin, Miller, Turk, & Hegarty, 2006; Michelon & Zacks, 2006) resulting in a U-shaped pattern. According to Kessler and Thomson (2010), the egocentric-specific RT pattern could be ascribed to the use of different strategies for small and large angular disparities. Whereas smaller angles seem to be solved with a visual matching process, larger angles evoke perspective transformations of the own body resulting in a higher mental effort and thus in higher RTs. Note that there are several further approaches to explaining differences in the angular disparity effect between egocentric and object-based transformations (cf. Parsons, 1987; Zacks, Mires, et al., 2002). 

### Developmental changes in mental rotation

 There is a huge body of evidence showing that two factors contribute to MR: 1) working memory and 2) processing speed (Booth et al., 1999; Hertzog & Rypma, 1991; Salthouse, Mitchell, Skovronek, & Babcock, 1989; Zacks, Mires, et al., 2002). 

#### Working memory

 MR involves several sub-processes (cf. Heil & Rolke, 2002). Prior to the actual rotational process the to-be-rotated stimulus must be encoded into memory. Subsequent to the rotation the imagined stimulus must be aligned with the comparison stimulus. Therefore, representations used in each particular sub-process must be maintained to enable access to information during the next stage. Even though there are only a few behavioral studies (Bruyer & Scailquin, 1998; Hyun & Luck, 2007) supporting the involvement of working memory (WM) in MR, the assumption that developmental change in MR reflects a deficit in WM should also be taken into account. 

 However, there is evidence that age differences in WM are mediated primarily by differences in information processing speed. For example, the relationship between age and WM was diminished after incorporating information processing speed as covariate (Hale & Jansen, 1994; Salthouse, 1991, 1992, 1994; Salthouse & Babcock, 1991). 

#### Processing speed

 According to the hypothesis of Birren (1974) increasing RTs are linked to age-related reductions of processing speed in the central nervous system (Cerella, 1985). Reduced information processing speed is also found in children (Kail, 1991). Since myelination increases the speed of information processing, slowing of information processing is attributed to a reduced myelination of axons in the central nervous system that only gradually matures during late childhood and adolescence from 4-17 years of age (Paus et al., 1999) and degrades with age from 78 years on (Meier-Ruge, Ulrich, Brühlmann, & Meier, 1992). 

 It is still unclear how processing speed and WM interact, but the resource-deficit hypothesis (Kahneman, 1973) could explain why age differences emerge when task difficulty increases. This hypothesis is based on the notion that increasing task difficulty has only a negative impact on performance when cognitive resources are limited. Both WM capacity (Salthouse, 1990) and processing speed (Birren, 1974; Salthouse 1998) fit the definition of cognitive resources. Since difficult tasks are resource-demanding, age differences could emerge due to a developmental or age-related decrease in the amount of resources. Taking into account that WM capacity and processing speed are cognitive resources (Baddeley & Hitch, 1974; Craik, 1977) reduced MR performance in children and the elderly could be ascribed to a developmental and age-related lower WM capacity or reduced processing speed. 

### Developmental changes in object-based and egocentric transformations

#### Childhood

 Regarding object-based rotations, Piaget and Inhelder (1971) claimed that visuo-spatial imagery appears only at the age of 8 years. However, there is some evidence for an earlier onset. For example, Estes (1998) observed 4-year-olds, 6-year-olds, and adults in an MR task in which participants had to decide whether two monkeys were holding up the same arm or different arms, resulting in an object-based transformation task. The results showed that already at the age of 4 years some children were both able to spontaneously use MR and be aware of this mental process when they were asked to explain the strategy they used. 

 Similar results are provided by Marmor (1975) who compared 5- to 8-year-olds in an object-based rotation task and showed similar patterns in RTs leading to the assumption that both age groups used MR in their visual imagery. However, Kail, Pellegrino, and Carter (1980) found this increase for 8-year old children only. Despite these contradicting results regarding the onset of a sufficient MR skill, there is a lot of evidence for a developmental change. For example, Kail et al. compared 3, 4, and 6 graders as well as college students and showed that MR speed nearly doubles between grades 3 and 4 (about 143°/s) and adults (about 250°/s). These findings led to the assumption that MR ability is subject to developmental changes. 

 According to Piaget and Inhelder (1971), who contrasted object-based and perspective rotations in two series of studies, children fail to solve egocentric transformations until they are 9-10 years of age, whereas rotation problems are solved already at the age of 7-8 years. However, it should be noted that both conditions are only comparable to a limited extent because of the divergent stimulus materials used. Therefore, Huttenlocher and Presson (1973) compared object-based and egocentric transformations under maximally similar conditions. Results showed a decreased performance in perspective rotations which is ascribed to the higher difficulty of the perspective task compared to object rotation. The researchers drew this conclusion on the basis of the results of two experiments. In the first experiment, the children were required to solve two different types of problems: 1) They had to describe the appearance of an array of objects that was rotated (rotation problem), and 2) children were required to anticipate the appearance of a fixed array to an observer being moved with respect to it (perspective problem). Children showed higher error rates in the perspective transformation. In the second experiment the children had to solve two types of perspective problems. In addition to the perspective task of the first experiment, children were required to describe the appearance of the array after they had moved around it. The latter was much easier to solve. The researchers concluded that the congruence between the observer and the own person is a contributing factor and that children are unable to integrate the perspective of a person which is not compatible with their own perspective. 

#### Adulthood

 A great deal of research has investigated whether there are performance differences in object-based and egocentric rotations. This research has established that egocentric transformations are solved faster and more accurately compared to object-based rotations (Amorim & Stucchi, 1997; Creem, Wraga, & Proffitt, 2001; Wraga et al., 2000; Wraga, Shephard, Church, Inati, & Kosslyn, 2005). 

 There are several suggestions as to how to explain this discrepancy. For example, Wraga et al. (2000) assumed different reference frames to be responsible for the different outcome of object-based and egocentric transformations. Another explanation for the RT advantage of egocentric over object-based transformations was provided by Zacks, Mires, et al. (2002). Since there is no image interference in left-right tasks, the visual buffer—as the neuronal substrate for both imaginal and perceptual visuospatial transformations—is not that highly loaded as it is the case in object-based transformations. However, this advantage of perspective transformations diminishes when the task requires imagining physically impossible rotations as was pointed out by Carpenter and Proffitt (2001), who argued for an embodied cognition approach. According to this view, better performance in egocentric transformation tasks is due to an enhanced activity in motor and motor-related structures through simulations of one’s own body underpinned by the activity of motor neurons, which do not occur in object-based transformations (Gallese, 2005). 

#### Senior age

 Regarding the investigation of age differences in object-based transformations, several researchers have demonstrated that older adults show slower responses and a lesser accuracy rate compared to young participants (Hertzog, Vernon, & Rypma, 1993; Kemps & Newson, 2005). Gaylord and Marsh (1975) revealed that MR speed was 84% slower than that of young adults. The reactions of older adults were slowed by a factor of 1.8 compared to the younger adults (9.6°/s vs. 17.7°/s), expressed by a stronger decrease of the slope relating rotation angle to RT for older adults. This is in line with the findings of Cerella, Poon, and Fozard ( 1981) who observed an age-decline of 96%. 

 Jansen and Kaltner (2014) investigated both types of transformations by comparing two object-based conditions (letters, human figures) with one egocentric transformation using a single human figure in participants ranging from 60 to 71 years. In this study, participants had to solve two (object-based) shape-matching tasks and one (egocentric) laterality judgment task. RT results showed that letters were solved faster compared to human figures in the laterality judgment task and in the shape-matching task, but no RT difference was found between the latter two conditions: body figure object (BFO) and body figure egocentric (BFE) task. Therefore, in older adults there was no egocentric-advantage regarding RTs. 

 Inagaki et al. (2002) investigated age-related differences in both transformation types by assessing ninety participants who were grouped into young (18-29 years), middle aged (30-59 years), and older adults (60 years and above). Whereas in the perspective transformation task an age-related decline was observed, no such decrease was found in the object-based transformation task. Herman and Coyne (1980) found a similar response pattern: Whereas hit rate in object-based rotations was not affected by age, performance in egocentric transformations decreased. However, Inagaki et al. as well as Herman and Coyne assessed solely accuracy as dependent variable while disregarding RT. Since both development (Rigal, 1996) and age-related decrease of cognition ( Briggs, Raz, & Marks, 1999) are reflected in this variable, it is also important to look at RTs. Therefore, Devlin and Wilson (2010) assessed both hit rate and RTs. They used letters, hands, and whole-body figures as stimuli and found that the decline was more pronounced for whole body stimuli (egocentric transformation) compared to hand stimuli and letters, specifically in object-based transformations. The authors assumed that the decline restricted to egocentric transformations could be ascribed to the difficulty to integrate information related to the body schema ( Devlin & Wilson, 2010). 

### Goals and hypotheses of the present study

The present study was conducted to provide a unique design which compares three different age groups concerning their performance in object-based and egocentric transformations. Neither the comparison of three age groups using standardized conditions nor the differentiation between these two types of transformations with a focus on developmental changes has been provided by previous research.

 Based on the findings that support both the involvement of WM processes in MR ( Bruyer & Scailquin, 1998; Hyun & Luck, 2007) and the evidence of impaired WM performance in both children (Gathercole, Pickering, Ambridge, & Wearing, 2004) and older adults (Dror, Schmitz-Williams, & Smith; 2005), we expected both groups to show increased RTs compared to adults (Hypothesis 1). 

 According to the complexity hypothesis of Cerella, Poon, and Williams (1980) increasing task difficulty leads to decreasing task performances in both children and older adults whereas this pattern is not observed in adults. This specific response pattern may be ascribed to limited cognitive resources of both children and older adults. Hereby, a reduced WM capacity of both groups (Gathercole et al., 2004; Salthouse et al., 1989) as well as a slowing in processing speed (Meier-Ruge et al., 1992) could be contributing factors. The better performance of adults may be due to an increase of information-processing speed during early adulthood or until the age-related decline in the elderly commences (Neimark, 1975). If this is true, a higher task difficulty in MR should affect children and older adults to a higher extent than adults. More specifically, we expected a steeper increase of RTs with increasing angular disparities in children and older adults compared to adults (Hypothesis 2). 

 Differences in object-based and egocentric transformations have been mostly investigated in adults (Amorim & Stucchi, 1997; Creem et al., 2001; Wraga et al., 2005). According to the literature (cf. experiment 1 of Huttenlocher & Presson, 1973; Jansen & Kaltner, 2014), we assumed no differences between object-based and egocentric rotations in children and older adults. That is, no egocentricity-advantage over object-based transformations is expected in the children and older adults group (Hypothesis 3). 

## Experiment

### Methods

#### Participants

 Sixty children, 31 boys and 29 girls, (age range: 8-11 years, *M*_age_ = 9.07, *SD* = .68), 73 adults, 36 men and 37 women (age range: 18-25 years, *M*_age_ = 23.48, *SD* = .78), and 62 subjects of older age, 31 men and 31 women (age range: 60-71 years, *M*_age_ = 65.87, *SD* = 3.99), participated in the study. However, gender differences were not taken into consideration because, on the one hand, an analysis of covariance showed that this factor had no influence on the results as well as no effects regarding stimulus material and group and, on the other hand, we wanted to focus exclusively on the developmental trajectory of rotation performance. Children were recruited from local schools; younger adults were recruited by advertisement at the university. The 62 older adults were randomly chosen from a former study investigating motor effects in MR (Jansen & Kaltner, 2014). The older participants received €10 for participation. None of the older adults showed a cognitive deficit, as measured with the Mini-Mental State Examination (MMSE) (Folstein, Folstein, & McHugh, 1975) and the Clock Test (Tuokko, Hadjistavropoulos, Miller, & Beattie, 1992). Participants or, for the children, their parents gave informed consent for participation.

#### Apparatus and Stimuli

**Figure 1.**
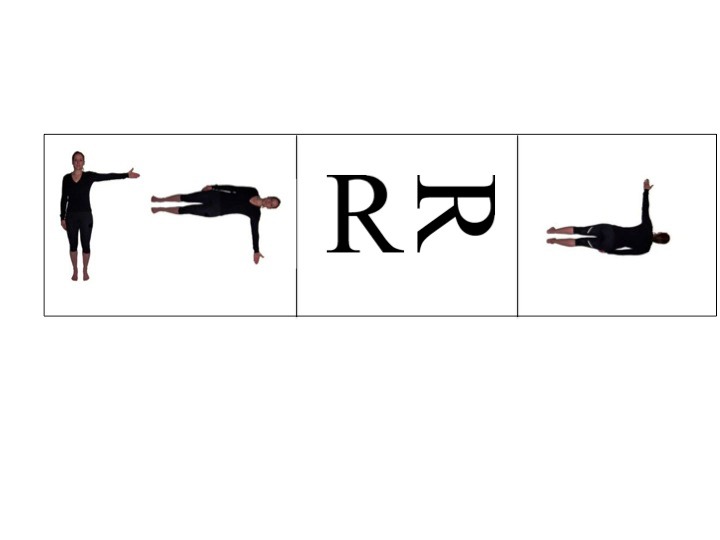
Examples of the three different stimulus types, a) body figures object-based (BFO), b) letters, and c) body figures egocentric (BFE).

*Mental rotation test*. MR performance was assessed by a chronometric mental rotation test (cMRT). Whereas the psychometrical paper and pencil version (Vandenberg & Kuse, 1978) solely assesses the accuracy, this test additionally provides RTs which are very important for analysing developmental aspects ( Briggs et al., 1999; Rigal, 1996). The cMRT consisted of three different stimuli, namely a) a frontal view of two women in black clothes who raised either the left or the right arm (BFO), b) the black letters R and F, and c) the front and back view of one women who raised either the left or right arm (BFE), see Figure 1. The test was presented on a laptop with a 17” monitor located approximately 60 cm in front of the participant. 

In the BFO and letter condition two stimuli were presented simultaneously with an angular disparity of 0°, 45°, 90°, 135°, or 180°. The left stimulus was always presented upright in the normal chirality. Half of the trials were pairs of identical objects and half were mirror-reversed images. In the BFE condition only one picture of a woman in black clothes who raised either the left or right arm was presented in the rotation angles mentioned above. Both in the BFO and in the BFE condition the to-be-compared images always showed the same female human figure. Besides, the BFE stimuli were from the same view (front or back) and the view only changed between trials and not within trials. The to-be-compared letters were always of the same identity. All stimuli were rotated in the picture plane. The order of the blocks was counterbalanced.

#### Procedure

 The individual test sessions which lasted about 60 min in total took place in a laboratory at the University of Regensburg for the older and younger adults and in a quiet room of the primary school for the children. Only older adults completed the MMSE (Folstein et al., 1975) and the Clock Test (Tuokko et al., 1992) at the beginning of the session. 

 The cMRT was conducted with a standardized task instruction. In both object-based conditions (BFO and letters) participants had to decide as quickly and as accurately as possible if the stimulus on the right side was identical (that is only rotated) to the comparison stimulus (shown on the left side)—we call it “same”, or if it was not identical—we call it mirror reversed (that is rotated plus mirrored) or “different”. If the stimuli were the same participants had to press the left mouse button (left-click) and they had to press the right mouse button (right-click) when the two stimuli were different. In the BFE condition, where only one picture of a woman in back- or front view was presented, participants had to decide which arm was raised. They had to press the left mouse button (left-click) when the figure raised the left arm and the right mouse button (right-click) when the right arm was raised. In the BFO condition the figures were presented solely in the front view. Presenting BFO figures in both front and back view would have resulted in more trials in the BFO condition compared to the BFE condition. In both conditions, we had two stimuli per block (BFO: left vs. right arm, BFE: front vs. back). This means that taking the view in the BFO condition into account by presenting the figures in both front and back view would have resulted in four stimuli per block in the BFO condition versus two stimuli per block in the BFE condition. This is not feasible for the analysis. Therefore, we decided to present the front view only in the BFO condition since there is evidence that the view is only relevant in a perspective transformation (Jola & Mast, 2005). 

At the beginning of each trial a fixation cross was presented for 1 s. After this, the pair of stimuli appeared and stayed on the screen until participants answered. Feedback was given for 500 ms after each trial in the middle of the screen with a “+” for a correct response and a “−” for an incorrect response. The next trial began after 1,500 ms. For each type of stimulus there was a separate block with 80 trials, each block was preceded by eight practice trials. After every 10 trials within each block a pause of 15 s was given before the next ten trials were administered. Between each of these blocks a break of around 1 min was taken. The presentation of the three blocks was randomized.

The experiment consisted of three blocks (BFE, letters, BFO) of 80 experimental trials each, resulting in 240 trials in total. The 80 trial were composed of 2 decision types (same/different vs. left/right) × 5 angular disparities (0°, 45°, 90°, 135° or 180°) × 4 repetitions of each combination × 2 types of stimuli (BFO: human figure where left vs. right arm was raised; letters: *R*, *F*; BFE: front vs. back view of the human figure ).

Thereby order of the presentation of the stimuli was randomized. For the BFE condition the responses for the women in front and back view were collapsed.

#### Statistical analysis

Two repeated-measures analyses of variance were conducted with stimulus, group, and angular disparity as independent variables and RT and accuracy rate as dependent variables. RT data were trimmed within subjects and means were taken. Data of eight children, two adults and three older adults had to be excluded because RT was higher than two *SD*s above the mean of the specific stimulus. Hereby, we filtered the data for the outliers separately for each age group. Error trials were not included in the analyses of RTs.

### Results

#### Mental Rotation: Reaction Time

Concerning RT, the analysis of variance showed three main effects for the variables stimulus, *F*(2, 384) = 69.73, *p* < .001, η_p_^2^ = .27, angular disparity, *F*(4, 768) = 628.21, *p* < .001, η_p_^2^ = .77, and group, *F*(2, 192) = 185.92, *p* < .001, η_p_^2^ = .67. Letters (*M* = 1,223 ms, *SD* = 556 ms) yielded a shorter RT than object-based (*M* = 1,553 ms, *SD* = 609 ms), *t*(194) = 8.52, *p* < .001, and egocentric human figures (*M* = 1,619 ms, *SD* = 717 ms), *t*(194) = -9.73, *p* < .001. There was no difference between the two conditions with the human figures, *t*(194) = 0.65, *p* = .515. Concerning the main effect of angular disparity, post-hoc comparisons showed that each angular disparity differed from the next smaller one (all *p* < .001). Children (*M* = 2,007 ms, *SD* = 515ms) showed a higher RT than adults (*M* = 865 ms, *SD* = 126 ms), *t*(131) = 18.29, *p* < .001, and older adults (*M* = 1,522 ms, *SD* = 308ms), *t*(120) = 6.33, *p* < .001, who in turn showed a significantly higher RT than adults, *t*(133) = -16.64, *p* < .001, see Table 1.

**Table 1.** Main Effect for the Variable “Group”

Group	*M* (ms)	*SD* (ms)
Children	2,007	515
Adults	865	126
Older adults	1,522	308

*Note*. *M* = mean reaction time, *SD* = standard deviations

Furthermore, there were three two-way interactions, 1) between stimulus and group, *F*(4, 768) = 8.49, *p* < .001, η_p_^2^ = .08, 2) between angular disparity and group, *F*(8, 1536) = 29.17, *p* < .001, η_p_^2^ = .23, and 3) between stimulus and angular disparity, *F*(8, 1536) = 8.28, *p* < .001, η_p_^2^ = .04.

**Figure 2.**
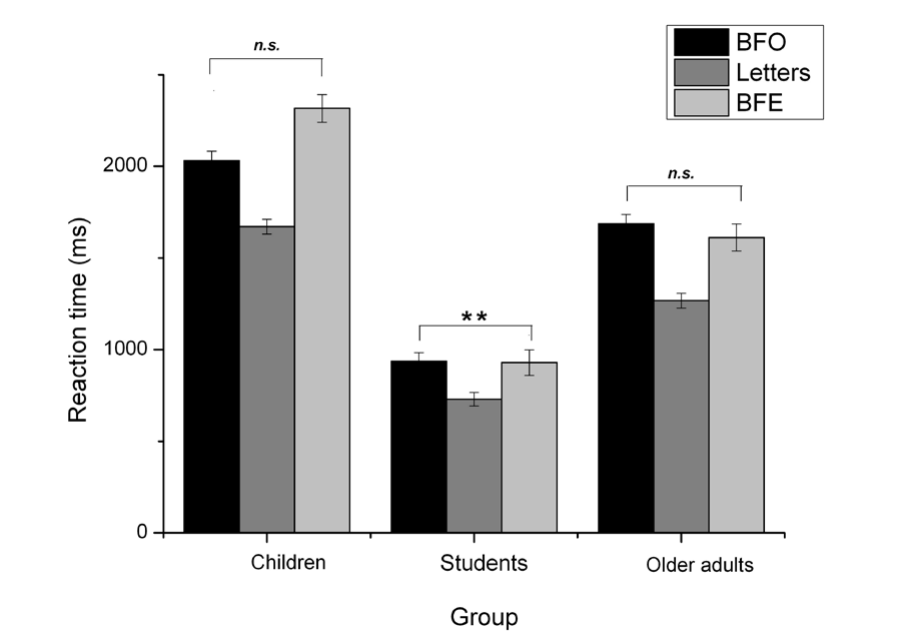
Mean reaction times and standard deviations (error bars) dependent on stimulus type and group. BFO = body figure object , BFE = body figure egocentric task.

1) The interaction of stimulus and group resulted from the fact that there was a significant difference between the object-based and egocentric human figure condition only within the adult group (BFO: *M* = 937 ms, *SD* = 167 ms; BFE: *M* = 929 ms, *SD* = 141 ms), *t*(72) = 3.58, *p* = .001, but not among children (BFO: *M* = 2,032 ms, *SD* = 511 ms; BFE: *M* = 2,316 ms, *SD* = 714 ms), *t*(59) = -1.62, *p* = .110, or older adults (BFO: *M* = 1,687 ms, *SD* = 450 ms; BFE: *M* = 1,612 ms, *SD* = 459 ms), *t*(61) = 1.75, *p* = .085, as shown in Figure 2. For a more detailed understanding, all means and standard deviations are given in Table 2.

**Table 2.** Results for the Stimulus × Group Interaction

		Interaction (ms)	
Group	Stimulus	*M*	*SD*
Children	BFO	2,032	511
	Letters	1,673	544
	BFE	2,316	714
Students	BFO	937	167
	Letters	729	124
	BFE	929	141
Older adults	BFO	1,687	450
	Letters	1,267	309
	BFE	1,612	459

*Note*. *M* = mean reaction time, *SD* = standard deviations, BFO = figures object-based, BFE = body figures egocentric

**Figure 3.**
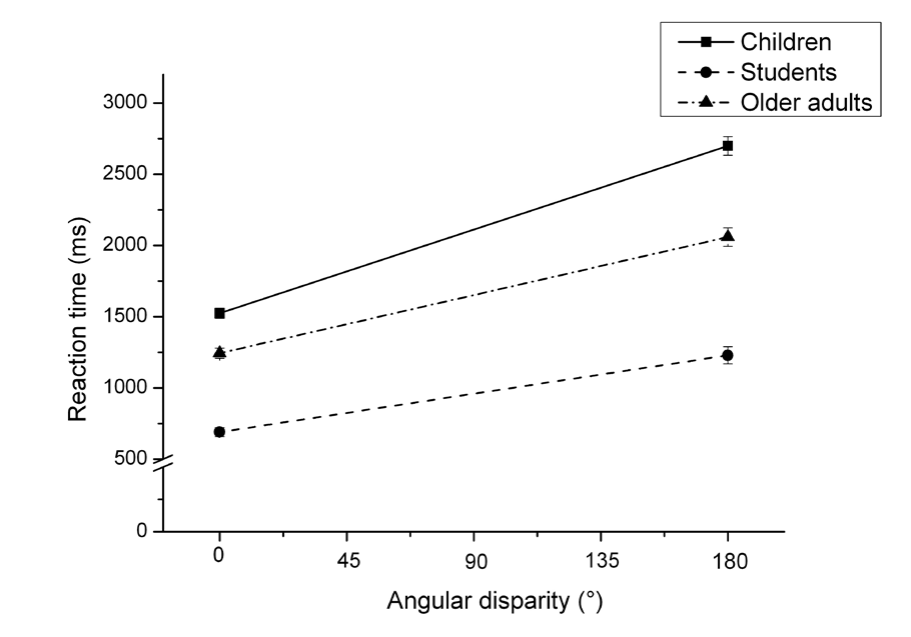
Mean reaction times and standard deviations (error bars) dependent on angular disparity and group.

**Figure 4.**
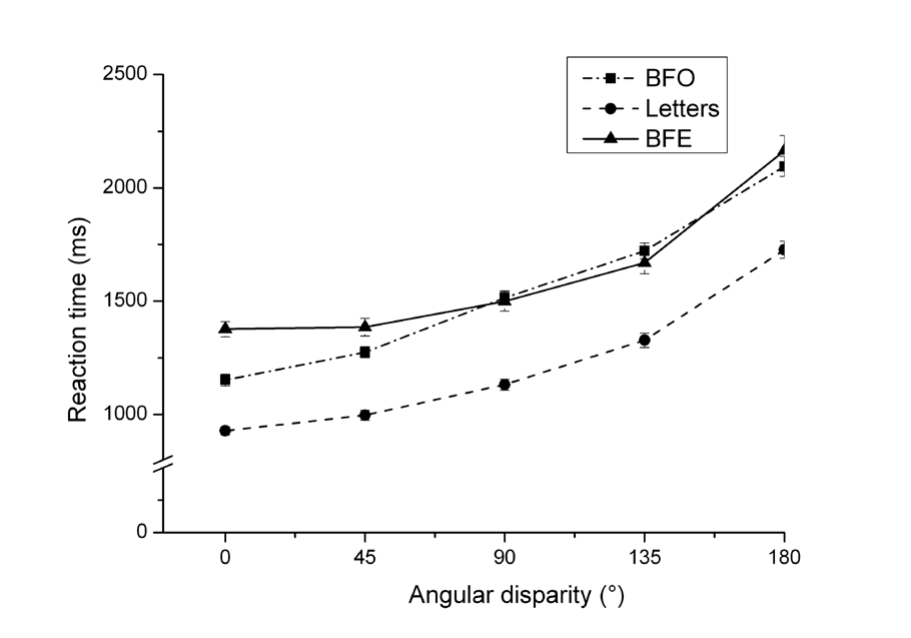
Mean reaction times and standard deviations (error bars) dependent on angular disparity and stimulus.

2) Regarding the interaction of angular disparity and group, post-hoc tests showed that the increase of RTs between 0° and 180° was significantly stronger in children (*M*_Diff_ = 1,088 ms, *SD* = 778 ms), *t*(131) = 6.66, *p* < .001, than in older adults (*M*_Diff_ = 626 ms, *SD* = 378 ms; *t*(120) = 4.19, *p* < .001), which was in turn significantly stronger than in adults (*M*_Diff_ = 408 ms, *SD* = 166 ms), *t*(133) = -4.45, *p* < .001. The increase of RTs in children was significantly stronger compared to that in adults, *t*(131) = 7.27, *p* < .001, see Figure 3.

3) Concerning the interaction between stimulus and angular disparity, RTs in the object-based conditions (letter, BFO) increased with increasing angular disparity, but they showed a U-shaped pattern for the egocentric transformation condition with human figures (BFE). This pattern was due to a significant difference between the RT of each angular disparity and the next smaller one in both object-based conditions (all *p* < .001), whereas no significant difference emerged between the angular disparities of 0° and 45° in the egocentric condition, *t*(194) = -.254, *p* = .800, as illustrated in Figure 4.

#### Mental Rotation: Accuracy

Concerning accuracy, the analysis of variance showed two significant main effects, for group, *F*(2, 192) = 23.05, *p* < .001, η_p_^2^ = .19, and for angular disparity, *F*(4, 768) = 122.04, *p* < .001, η_p_^2^ = .39. Besides, two significant interactions emerged, between angular disparity and group, *F*(8, 1536) = 4.50, *p* < .001, η_p_^2^ = .05, as well as between stimulus and angular disparity, *F*(8, 1536) = 11.24, *p* < .001, η_p_^2^ = .05.

Bonferroni-corrected *t*-tests regarding the main effect of group showed a significantly lower accuracy for the children (82.3%, *SD* = 12.5) than for the adults (92.1%, *SD* = 5.8), *t*(131) = -5.94, *p* < .001, and the older adults (90.8%, *SD* = 7.2), *t*(120) = -4.62, *p* < .001. There was no significant difference between the adults and older adults, *t*(133) = 1.15, *p* = .254, see Table 3. This table also includes the RTs and accuracies split for all three variables, group, stimulus, and angular disparity.

**Table 3.** Main Effects of the Variables Group, Stimulus, and Angular Disparity

Factor		Reaction time (ms)		Accuracy (%)	
		*M*	*SD*	*M*	*SD*
Group	Children	2,007	515	82.3	12.5
	Students	856	126	92.1	5.8
	Older adults	1,522	308	90.8	7.2
Stimulus	BFO	1,553	609	88.1	13.3
	Letters	1,223	556	89.8	18.5
	BFE	1,619	717	87.3	19.1
Angular disparity	0°	1,152	473	92.6	8.1
	45°	1,219	480	91.5	9.6
	90°	1,382	559	90.9	10.7
	135°	1,573	639	88.3	11.8
	180°	1,995	880	78.7	16.5

*Note*. *M* = mean, *SD* = standard deviations

Regarding the main effect of angular disparity, Bonferroni-corrected *t*-tests revealed that accuracy decreased significantly between the angular disparities of 90° and 135°, *t*(194) = 4.57, *p* < .001, and between 135° and 180°, *t*(194) = 11.17, *p* < .001. There was no significant difference in the accuracy between angular disparities of 0° and 90° (0°-45°: *t*[194] = 2.12, *p* = .035; 45°-90°: *t*[194] = 1.26, *p* = .208).

Concerning the interaction between angular disparity and group, it was shown that, compared to the adult group (*M*_Diff_ = 9.4%, *SD* = 11.3), the decrease of accuracy between the angular disparities of 0° and 180° was significantly stronger in the children group (*M*_Diff_ = 14.6%, *SD* = 14.93), *t*(131) = -2.29, *p* = .001, and in older adults (*M*_Diff_ = 17.8%, *SD* = 15.42), *t*(133) = 3.63, *p* = .010. Children and older adults did not differ significantly, *t*(120) = 1.15, *p* = .713.

The interaction between stimulus and angular disparity resulted from the fact that the decrease of accuracy between angular disparities of 0° and 90° was stronger for the letters (*M*_Diff_ = 19.61%, *SD* = 26.80) than for the BFO stimuli (*M*_Diff_ = 11.35%, *SD* = 20.55), *t*(194) = 4.06, *p* < .001, and for the BFE stimuli (*M*_Diff_ = 10.01%, *SD* = 16.44), *t*(194) = -4.22, *p* < .001. The latter two conditions did not significantly differ, *t*(194) = -0.73, *p* = .468.

Further analysis showed that the mean RT was negatively correlated with the accuracy rate, *r* = -.488, *p* < .001. This does not hold true for children, *r* = -.185, *p* = .157, but for adults, *r* = -.652, *p* < .001, and older participants, *r* = -.634, *p* < .001.

### Discussion

A great deal of research addressed MR performance of different age groups, like children, adults, and older adults. However, little is known about the difference between object-based and egocentric transformations with a focus on their developmental change. This was the main issue of the present study. Important results were that children and older adults showed slower overall RTs compared to adults, confirming Hypothesis 1. Regarding the RT pattern, with increasing task difficulty, children and older adults showed a steeper increase of RTs with increasing angular disparity compared to adults, which provides evidence for Hypothesis 2. Interestingly, the children showed both higher overall RTs, a lesser accuracy and a steeper increase of RTs with increasing angular disparity compared to older adults. With respect to the types of transformations, the comparison of RTs in object-based and egocentric transformations revealed that only in adults there was a difference between BFO and BFE stimuli expressed by higher RTs for BFO stimuli, which did not occur for both children and older adults. This finding corroborates Hypothesis 3.

#### Developmental changes in mental rotation

Results concerning hypotheses 2 and 3 have in common that children and older adults showed decreased task performance compared to adults. They showed higher overall RTs as well as a steeper increase of RTs with increasing task difficulty. Both results can be interpreted by developmental and age-related differences in the following contributing factors: 1) WM, and 2) processing speed.

#### Working memory

 The involvement of WM processes in MR is supported by Booth et al. (1999) who demonstrated that mentally rotated stimuli were temporally stored in WM. Furthermore, Gathercole et al. (2004) claimed that especially the visuo-spatial sketchpad, a subsystem of the WM, plays an important role for the manipulation of visual images. The idea of an involvement of the visuo-spatial sketchpad in MR is supported by the results of a study by Lehmann, Quaiser-Pohl, and Jansen (2014) . The researchers revealed a positive correlation between spatial WM capacity measured by the Corsi block tapping task and mental rotation performance. However, it should be noted that it is still an open question whether other parts of the WM or only specific components such as the visuospatial sketchpad are involved in MR. In this context, Shah and Miyake (1996) underlined the separability of spatial and verbal WM resources for spatial thinking and further revealed that both the processing and storage components of WM tasks are important for predicting spatial thinking performance. 

 Gathercole et al. (2004) provided substantial evidence that WM undergoes an important developmental shift during early school years, ascribed to assumed increases in storage capacity or deployment of strategies. Results showed that the basic tripartite model of WM of Baddeley and Hitch (1974), consisting of phonological loop, central executive, and visuospatial sketchpad, develops from 4 years onward. Considerable research investigated the increase of WM ability from childhood to adulthood was investigated largely (Chelonis, Daniels-Shawb, Blakea, & Paule, 2000; Conklin, Luciana, Hooper, & Yarger, 2007; Kemps, De Rammelaere, & Desmet, 2000) and this development was attributed to a greater activation in frontal, parietal, and cingulate regions, known to support WM performance (Kwon, Reiss, & Menon, 2002; Schweinsburg, Nagel, & Tapert, 2005). These developmental changes may contribute to the RT differences between children and adults found in the present study. Similarly, the impaired RT performance of older adults could also be explained by a decline in WM ability found by Hertzog and Rypma (1991) . The authors demonstrated an age-related loss of visuo-spatial information from WM when MR was required. 

#### Processing speed

 There is some evidence that MR speed undergoes an important developmental shift from childhood to adulthood, and declines with increasing age (Jansen & Kaltner, 2014; Kail, 1991; Kail et al., 1980; Neimark, 1975). Researchers argued that cognitive aging is caused by a general decrease in information-processing speed (Birren, 1974; Hertzog & Rypma, 1991). For example, Lindenberger, Mayr, and Kliegl (1993) showed that processing speed predicted age-related differences in intellectual abilities beyond 70 years of age. Therefore, age differences in intelligence among old and very old adults could be mediated by age differences in speed. Similar results were provided by Fry and Hale (1996) for the age-related increase in fluid intelligence from children to adolescents and young adults (7 to 19 years). Half of the increase in this intellectual ability was mediated by developmental changes in processing speed and WM. 

 Considering this special relationship between cognitive processing speed and WM, it still remains unclear, however, whether the age-dependent RT differences in the present study were mediated by developmental changes in processing speed or WM capacity. However, there is evidence that age differences in WM are mediated primarily by differences in information processing speed. For example, the relationship between age and WM was diminished after assessing information processing speed as covariate (Hale & Jansen, 1994; Salthouse, 1991, 1992, 1994; Salthouse & Babcock, 1991). In line with this literature, information processing speed is a global construct which should be taken into account in the interpretation of MR results. Therefore, we conclude that higher RTs of children and older adults as well as a steeper increase of RTs with increasing angular disparity in both groups can also be mediated by age differences in processing speed. 

 Interestingly, our results further revealed that the children showed slower overall RTs and a lesser overall accuracy as well as a steeper RT increase with increasing task difficulty compared to older adults. This leads to the assumption that children of the age range assessed in the present study were not comparable with older adults regarding their developmental or age-related changes in MR performance, processing speed, or WM capacity. That is, the assumption of an inverted U-shaped pattern of cognitive development proposed in the literature (Conklin et al., 2007; Gathercole et al., 2004, Hertzog & Rypma, 1991; Jansen & Kaltner, 2014; Neimark, 1975; Kail, 1991; Kail et al., 1980) has to be investigated in further detail. Future work should include a large variety of age ranges both in children and older adults. 

#### Developmental changes in object-based and egocentric transformations

 Although there are a handful of studies that have dealt with developmental changes in MR performance (Kail et al., 1980; Kosslyn, Margolis, Barrett, Goldknopf, & Daly, 1990; Gaylord & Marsh, 1975), so far little was known about developmental changes of the two types of transformations in MR, namely object-based and egocentric rotations. The investigation of developmental changes with a focus on this differentiation between object-based and egocentric transformations was the main issue our study sought to address. Analyses showed that whereas RTs did not differ between object-based and egocentric human figures in children and older adults, adults needed longer to solve BFO stimuli compared to BFE human figures. 

 The performance advantage of egocentric transformations over object-based rotations in the adults group of the present study is in line with previous literature (Amorim & Stucchi, 1997 ; Creem et al., 2001; Wraga et al., 2000, 2005). However, this performance advantage of egocentric transformations could not be revealed for children and older adults. We tentatively propose that the absence of an egocentric advantage in children and older adults could be interpreted as decreased performance restricted to this kind of transformation. 

 The idea of a decreased performance of children in the egocentric transformation task is in line with the work of Piaget and Inhelder (1971) who assessed children in the age between 9 and 11 years of age. Besides, it supports the findings of older adults provided by several studies (Devlin & Wilson, 2010; Jansen & Kaltner, 2014). 

 For example, Piaget and Inhelder (1971) revealed that children failed to solve egocentric transformations until they were 9-10 years of age, whereas rotation problems were solved already at the age of 7-8 years. The lower performance of children in egocentric transformations is also well known in developmental psychology and in line with the egocentrism postulated by Piaget (1926) . Here, Piaget noted that the so called „self-centeredness“ is expressed in the fact that children are not able to change their own perspective, as assessed by the mountain task (Piaget & Inhelder, 1956). Only children at the concrete operational stage at age 7 to 12 began to solve perspective-taking problems. This is in line with the results of Piaget and Inhelder (1971 ) . In contrast to their work, we held stimulus material constant which underlines the assumption that the transformation type itself is crucial in children. To investigate which mechanisms are responsible for an impaired egocentric performance in children further research is needed. Using neuroimaging studies could be a helpful approach due to the fact that distinct neuronal activations underlie both types of transformations: Whereas object-based transformations seem to be associated with right hemisphere activation, egocentric transformations primarily activate areas in the left hemisphere (Thakkar, Brugger, & Park, 2009). Similar results were provided by lesion studies: Ratcliff (1979) reported selective impairments in object-based transformations after lesions to the right posterior cortex, whereas lesions to the left posterior cortex led to problems when the participants were required to imagine themselves turning in a navigation task (Semmes, Weinstein, Ghent, & Teuber, 1963). The comparison of the neuronal activity between children, adults, and older adults could provide further useful information to clarify this issue. 

 Devlin and Wilson (2010) claimed that the decline in an egocentric transformation task might be due to the difficulty of integrating information relevant for the body schema. The body schema integrates “information about the position and extent of the human body (...) and therefore represents a spatiomotor representation of the body” (p.182, Buxbaum, Giovannetti, & Libon, 2000). Since egocentric transformations recruit the representation of the own body ( Parsons, 1994) the body schema seems to play an important role in egocentric transformations. In older adults, it was shown that the noise of neuronal signals from sensorimotor areas (e.g., posterior parietal cortex) increases with age which leads to a decreased ability to integrate information in order to build a stable representation of the own body (Ghafouri & Lestienne, 2000). In children, there are multiple evidences for a deficient body schema. For example, Schlater, Baker, and Wapner (1974) demonstrated an underestimation of the length of the arm in children from 7-18 years of age, whereas the size of the head was overestimated (Wapner, 1964). Furthermore, it was shown that the accuracy of estimations increases with age. Based on these findings, developmental changes in body schema should also be taken into account in the interpretation of children’s reduced ability to perform egocentric transformations.

### Limitations and Conclusion

Considering WM and processing speed, these variables should be assessed additionally to draw conclusions regarding their potential influence on MR performance.

Regarding methods, our study was limited by the fact that the sample of children ranged from age 8 to 11 years. Especially in this age group, literature is inconsistent regarding developmental changes in MR performance. Furthermore, the investigation of younger children could help to clarify the question of the onset of MR ability since previous literature is inconsistent because of several reasons mentioned above.

 Beyond that, it has to be noted that the direct comparison between egocentric and object-based transformations should be reconsidered in view of the fact that these types of transformations differ in several aspects: visual stimulation (2 stimuli vs. 1 stimulus, cf. Zacks, Ollinger, Sheridan, & Tversky, 2002), type of judgment (same-different vs. left-right, cf. Steggemann et al., 2011 ), and instruction (Borst, Kievit, Thompson, & Kosslyn, 2011), resulting in different stimulus presentations and response requirements. These confounding factors should be taken into account in future research. Especially regarding the two tasks using body stimuli (BFO, BFE), future analyses should examine age differences in two separate tasks due to inherent differences in instructions and judgments. Note that the interactions with transformation type observed in the current study were not affected by this consideration, however. 

 A further critical issue is the fact that in the BFE condition the factor “view” has an impact on the response pattern: In egocentric transformations a pattern of increasing RTs with increasing angular disparities is restricted to the back view. This means that away-facing figures (back view) were found to produce linear increases in RT with increasing rotation angle, whereas toward-facing figures were found to produce basically flat functions (Jola & Mast, 2005). This finding confirms the work of Zacks, Ollinger, et al. (2002) who found that performance for body figures in front view did not vary as a function of rotation angle. This inherent difference between front and back view should be taken into consideration in future work. 

 The present study was conducted to investigate developmental changes of MR performance with a focus on two types of strategies: object-based and egocentric transformations. So far, the development of the two types of transformations has not been a matter of research. In summary, this study revealed two important findings: 1) the role of an age-related decline in processing speed and with the possible importance of WM capacity in MR performance; 2) the observation that children and older adults seem to show deficits in perspective taking compared to adults. This finding supports previous work (Devlin & Wilson, 2010; Piaget & Inhelder, 1971) but sticks out by using a standardized design for each age group assessed. Therefore, we tentatively propose that perspective transformations are more sensitive to developmental change compared to object-based transformations. This leads to the remaining question as to when perspective transformations are exactly required and start to decline during the lifespan. This study provides a first step to investigate this issue, but further steps need to be taken in future research. 
